# A213 REDEFINING MILD-MODERATE ULCERATIVE COLITIS: PATIENTS WITH ENDOSCOPIC MAYO SCORE 1 AND ACTIVE HISTOLOGIC INFLAMMATION HAVE SIMILAR OUTCOMES TO PATIENTS WITH MAYO SCORE 2

**DOI:** 10.1093/jcag/gwae059.213

**Published:** 2025-02-10

**Authors:** N Khan, E Wong, N Narula

**Affiliations:** Internal Medicine, McMaster University, Hamilton, ON, Canada; Internal Medicine, McMaster University, Hamilton, ON, Canada; Internal Medicine, McMaster University, Hamilton, ON, Canada

## Abstract

**Background:**

Mild-to-moderate ulcerative colitis (UC) is conventionally defined as a total Mayo Clinic score of at least 4, with a Mayo Endoscopic Score (MES) of 2 and a Rectal Bleeding Score (RBS) of at least 1. Historically, endoscopic improvement with treatment has been defined as MES 0 or 1. However, studies have shown differences in prognosis between these two subsets of patients, with those who attain MES 1 reported to have higher rates of relapse. Patients with MES 1 and histologically active disease are currently excluded from contemporary clinical trials based on prevailing inclusion criteria. Consequently, insight into improving therapeutic outcomes for this patient population remains limited.

**Aims:**

This study aims to explore whether UC patients with lower endoscopic burden but active histology have comparable outcomes to those with “conventional” mild-moderate UC.

**Methods:**

This was a post-hoc analysis from the VARSITY study. Patients who completed induction (at week 14) with mild-moderate UC based on the conventional definition were compared to patients with histologically active MES 1 for achieving histo-endoscopic mucosal improvement (HEMI) at week 52, defined as Mayo endoscopic subscore ≤ 1 and Geboes highest grade < 3.2. Secondary outcomes included endoscopic remission (ER) (Mayo endoscopic subscore of 0), histologic improvement (Geboes highest grade < 3.2) and clinical remission (CR) (total Mayo score ≤ 2 and no subscore >1 on any of the four subcomponents). Histologically active disease was defined as Geboes highest grade > 3.2 (>50% of neutrophil crypt involvement in the epithelium).

**Results:**

Week 52 outcomes were comparable among patients with mild-moderate UC compared to those with histologically active disease and MES of 1. At week 52, a similar proportion of patients achieved HEMI [19/79 (24.1%) vs. 28/113 (24.8%), p=0.908], ER [23/79 (29.1%) vs. 35/113 (31.0%), p=0.782], histologic improvement [23/79 (29.1%) vs. 36/113 (31.9%), p=0.685] and CR [38/79 (48.1%) vs. 66/113 (58.4%), p=0.158].

**Conclusions:**

Disease outcomes in patients with MES 1 and a Geboes score > 3.2 were comparable to those with conventional mild-to-moderate UC. These results highlight the need for clinical trialists to revisit and expand the current definition of mild-to-moderate UC. Further validation of these findings in other patient cohorts is recommended.

**Funding Agencies:**

None

